# Artificial intelligence in orthopaedics: Enhanced examinations, ambient intelligence and the future of clinical practice

**DOI:** 10.1002/ksa.70339

**Published:** 2026-02-18

**Authors:** Alexander Bouterse, James A. Pruneski, Felix C. Oettl, Balint Zsidai, Thomas Tischer, Umile Giuseppe Longo, Romain Seil, Michael T. Hirschmann, Kristian Samuelsson

**Affiliations:** ^1^ Department of Orthopaedic Surgery Tripler Army Medical Center Honolulu Hawaii USA; ^2^ Department of Orthopaedic Surgery, Balgrist University Hospital University of Zürich Zurich Switzerland; ^3^ Sahlgrenska Sports Medicine Center Gothenburg Sweden; ^4^ Department of Orthopaedics, Institute of Clinical Sciences, Sahlgrenska Academy University of Gothenburg Gothenburg Sweden; ^5^ Department of Orthopedics Skåne University Hospital Malmö/Lund Sweden; ^6^ Department of Orthopaedic Surgery Universitymedicine Rostock Rostock Germany; ^7^ Department of Orthopaedic and Trauma Surgery Malteser Waldkrankenhaus Erlangen Erlangen Germany; ^8^ Fondazione Policlinico Universitario Campus Bio‐Medico, Via Alvaro del Portillo, 200 Roma Italy; ^9^ Research Unit of Orthopaedic and Trauma Surgery Department of Medicine and Surgery, Università Campus Bio‐Medico di Roma, Via Alvaro del Portillo, 21 Roma Italy; ^10^ Department of Orthopaedic Surgery Centre Hospitalier de Luxembourg Luxembourg City Luxembourg; ^11^ Kantonsspital Baselland University Department of Orthopaedic Surgery and Traumatology Bruderholz Switzerland; ^12^ University of Basel Basel Switzerland; ^13^ Department for Orthopaedics Sahlgrenska University Hospital Mölndal Sweden

**Keywords:** artificial intelligence, deep learning, machine learning, orthopaedics

## Abstract

Artificial intelligence (AI) continues to rapidly transform the practice of medicine, with clinicians increasingly adopting data‐driven decision‐making aids and diagnostic support tools. Orthopaedic physicians are well poised to harness the capabilities of AI, with an abundance of quantifiable imaging, biomechanical data, and structured clinical parameters lending themselves to algorithmic interpretation and automation. Namely, AI‐augmented vision systems may increase the breadth of information readily available to clinicians, whereas smart exam rooms and automated clinical summaries may soon streamline clinical workflows to decrease administrative burden and allow more time for direct patient care. Personalised education materials and visual aids may improve patient understanding and compliance, with the aim of optimising patient outcomes. Generative medical and orthopaedic event models may soon alter decision‐making heuristics and improve patient counselling. While the widespread adaptation of AI into clinical practices is not without limitations, physicians will likely come to share an increasingly symbiotic relationship with these platforms throughout their continued evolution. Accordingly, it is imperative that current and future orthopaedic practitioners become well‐versed in harnessing the capabilities of AI and continue to identify new avenues for such technologies to benefit clinicians and patients alike. As such, the current manuscript provides a narrative review of the potential future applications of AI within orthopaedic practices by exploring current and developing technologies and detailing how the continued integration of AI‐powered systems may serve to revolutionise the delivery of orthopaedic care.

**Level of Evidence:**

Level V.

**Abbreviations**
AI: artificial intelligence
AR: augmented reality
COMET: Cosmos Medical Event Transformer
DL: deep learning
EHR: electronic health record
LLM: large language models
ML: machine learning
NLP: natural language processing
RGB‐D: red‐green‐blue‐depth

## INTRODUCTION

The ongoing integration of artificial intelligence (AI) into healthcare systems has rapidly transformed the practice of medicine. As physicians increasingly trend toward the adoption of data‐driven support tools, AI presents a promising aid to clinical decision‐making, allowing for improved diagnostic accuracy, optimised treatment strategies, and individualised risk assessments [12, 21, 30, 42, 49, 55, 74]. Amongst clinicians, such tools are quickly becoming an essential part of clinical practice, with a recent large‐scale survey conducted by the American Medical Association indicating that more than two‐thirds of physicians have already integrated AI into aspects of patient care and that the majority of respondents see definitive advantages stemming from the use of AI‐powered tools [4]. Concurrently, a recent sample of more than 2,400 hospitals across the United States found that nearly 70% report active utilisation of AI to facilitate personalised risk stratification, guide longitudinal follow‐up, and manage administrative tasks [52].

Within the last decade, the number of orthopaedic publications pertaining to the use of AI has increased drastically, with clinical applications spanning from image evaluation and surgical planning to outcome prediction and advanced decision‐making [59, 60]. Accordingly, more than half of orthopaedic surgeons anticipate that AI will substantially influence their preoperative planning and documentation practices over the next decade and nearly 95% of orthopaedic clinicians report at least rudimentary skill in utilising AI [63]. The purpose of the current work is to provide a narrative review of the potential applications of AI within orthopaedic practices, exploring current and developing technologies and how the continued integration of AI‐powered systems may serve to revolutionise the delivery of orthopaedic care.

### AI‐supported vision systems

One of the more accessible and imminent uses of AI within orthopaedic clinical practice is the incorporation of real‐time AI support into commercially available eyewear/vision technologies. While existing augmented reality (AR) platforms, such as Microsoft HoloLens (Microsoft Corporation, Redmond, WA) or Google Glass (Google LLC, Mountainview, CA) smart glasses, can follow simple commands and display clinically relevant imaging and documentation, the enhancement of these technologies with AI software has the potential to improve diagnostic accuracy and streamline integration into the electronic health record (EHR). For instance, equipping clinicians with AI‐supported AR glasses during clinical examinations may allow for immediate quantitative measurements and recognition of common orthopaedic ailments, including abnormal gait patterns, deviations from anatomic limb alignment, and objective limitations in range of motion. While these assessments have not been specifically validated using clinician‐worn vision systems, machine learning (ML) and deep learning (DL) models have shown robust performance in independently performing assessments of gait, limb alignment, and joint motion, indicating that these functions are readily translatable to AI‐enabled eyewear platforms [41, 43].

The utility of AI‐powered vision technologies also extends into the operating room, with integration already occurring in an array of practice settings [14, 45]. The benefits of wearable surgical navigation aids are well‐documented, however, with further enhancement through AI software, critical intraoperative tasks, including real‐time measurement of anatomic landmarks, analysis of radiographic imaging, and localisation of surgical instrumentation, may become increasingly accurate and efficient [18, 20]. Proprietary medical smart glasses, such as the Vuzix M400 (Vuzix Corporation, Rochester, NY) and NextAR (Medacta International, Castel San Pietro, Switzerland) platforms, employ ML and DL algorithms to guide intraoperative workflows that may yield shorter operative times, reduce fluoroscopic exposure, and improve alignment of implanted devices [19, 27, 61, 64]. As these modalities become more commonly utilised as surgical aids, ongoing advancements in AI algorithms and sensor technologies are likely to enhance their precision and adaptability, potentially facilitating their extension beyond the operating room into clinical examinations, outpatient monitoring, and personalised treatment planning across orthopaedic subspecialties.

### Smart examination rooms and AI‐facilitated clinical encounters

**Figure 1:**
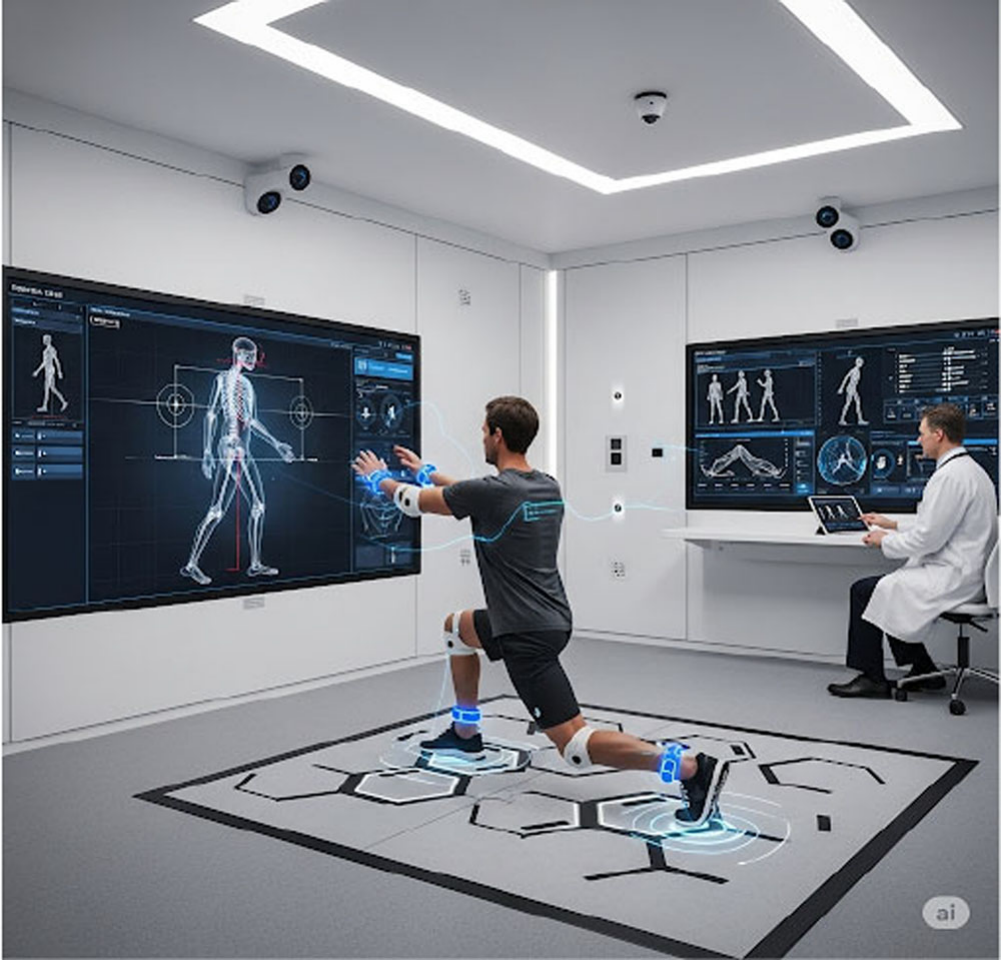
Conceptual design for a smart clinical examination room. Figure created with the assistance of OpenAI's DALL·E for illustrative purposes.

An extension of AI‐supported vision systems is the concept of a “smart examination room.” In this proposed environment, orthopaedic clinic rooms would be fitted with pressure‐ and motion‐sensitive technologies, Red‐Green‐Blue‐Depth (RGB‐D)‐capable cameras, and haptic and proprioceptive sensors, all of which would document and analyse inputs generated by the patient during guided movements or examination manoeuvres (Figure 1). Using the cumulative data from these devices, physicians could leverage objective measurements to support a clinical diagnosis, track recovery after surgery, or receive prompts to perform further targeted exams to confirm a potential diagnosis. Of note, ML and DL analyses of RGB‐D video recordings have been used to accurately categorise altered gait patterns and quantify minute limitations to active and passive range of motion [9, 38, 46]. Similarly, DL has been applied to data from pressure‐sensitive mats to detect subtle postural asymmetry, allowing for targeted interventions and therapy to prevent worsening pain and deformity [6]. Combining these technologies into a single, all‐purpose examination environment could greatly improve diagnostic efficiency by reducing the subjectivity inherent in many orthopaedic tests. While this concept is in its infancy, it presents an exciting opportunity to provide more accurate, objective, and reproducible assessments.

A more radical use of these technologies could also allow clinicians to make clinical encounters and diagnosis entirely virtual, with patients carrying out instructed movements while utilising at‐home devices equipped with the requisite diagnostic software. While this approach may never fully replace in‐person visits, it could supplement virtual encounters with objective data, thereby markedly improving the accuracy and utility of evaluations for those with geographic, socioeconomic, or functional limitations that preclude an in‐person examination. This strategy has already been enacted in a limited capacity, with one example including the use of ML to analyse gait patterns in postoperative patients equipped with wearable devices during at‐home ambulation [32, 75]. While undoubtedly there are financial and logistic barriers to more widespread implementation of this proposed method, it certainly carries significant potential for future orthopaedic practices and may be a catalyst for reframing the way orthopaedic evaluations are performed.

### Ambient intelligence and multimodal electronic health records

One of the more desired implementations of AI is the development of tools that assist with or optimise clinical documentation. With the advent of digitised medical records, the volume of data presented to physicians, and subsequently the amount of time devoted to accurately transcribing this information, has risen exponentially [3, 8, 37, 58]. Accordingly, over 74% of surveyed physicians currently report clinical documentation as a primary source of burnout and career dissatisfaction [13, 62].

The most promising solution to this crisis is the introduction of AI‐powered ambient intelligence platforms—discrete devices equipped with natural language processing (NLP) software to allow automated transcription of clinical encounters into the EHR with minimal input from the physician. Early implementation of this technology has already produced marked improvement in clinical efficiency with several platforms yielding reductions to note‐writing time, after‐hours documentation, and overall encounters times—all of which have correlated with improved job satisfaction, decreased mental demand, and declining metrics of burnout amongst clinicians [2, 7, 28, 68]. Furthermore, incorporation of large language models (LLMs) into EHR systems has demonstrated the ability of AI to outperform physicians when tasked with summarising and transcribing clinical text [31, 71, 78]. Within orthopaedics, the introduction of NLP and LLM platforms presents immense opportunity for increasing the speed and accuracy of clinical documentation. Particularly, AI tools could be leveraged to automatically generate detailed notes, templated physical exams, and injury‐specific documentation with minimal manual input, allowing orthopaedic clinicians to devote more time to patient care while ensuring standardised, high‐quality records across clinical settings. While the risk of inaccurate interpretation remains a risk with the utilisation of such platforms, particularly in the setting of multi‐lingual or cross‐cultural encounters, continued refinement and responsible integration of these tools promise to increase their utility and accuracy for current and future physicians.

**Figure 2:**
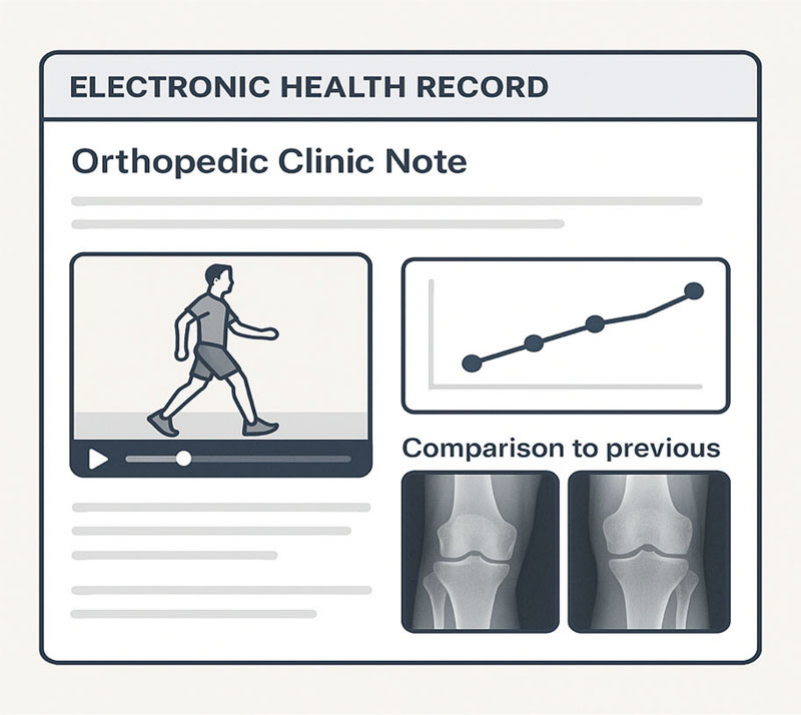
Future electronic health record (EHR) concept with embedded videos, graphic/tabular representations of clinical data (i.e., changes to ROM over time), and automatically generated comparisons of current and previous imaging studies. Figure created with the assistance of OpenAI's DALL·E for illustrative purposes.

Additionally, the adaptation of AI‐enabled EHRs would allow physicians to enhance clinical documentation by providing a more intuitive and comprehensive description of patient encounters. In orthopaedics specifically, clinicians may better characterise a patient's unique presentation in their notes through streamlined inclusion of detailed photographs, video analyses, and sensor array inputs (Figure 2). Furthermore, AI‐powered EHRs could act as an assistant to clinical decision‐making, prompting appropriate diagnostic orders, interdisciplinary referrals, and procedural interventions based on data recorded throughout the encounter.

### AI‐generated patient summaries and visual aids

Beyond increasing physician efficiency with documentation, the integration of AI into the EHR may also improve patients' comprehension of their pathologies and treatment plans. Specifically, AI‐generated summaries of clinical encounters and diagnostic results have illustrated greater inclusion of patient‐friendly language relative to those written by clinicians, while being less likely to omit important information and requiring a fraction of the time to create [23, 65]. The use of AI to assist with clinical summaries and patient education materials has been positively associated with enhanced longitudinal outcomes in orthopaedics, making AI a promising avenue for improving quality of care [24, 29, 40, 47, 70].

Alongside clinical summaries, visual teaching aids and communication tools have become an increasingly important component of patients' understanding of their treatment plan [35]. Within orthopaedics, this approach has been broadly beneficial, with patients reporting reduced levels of periprocedural anxiety and enhanced treatment protocol compliance when supplied with visual education materials [11, 22, 67]. Through further enhancement of these tools with AI, patients may benefit from more personalised, interactive, and comprehensible explanations of their diagnosis and treatment. For instance, automatically constructed three‐dimensional models of the structures involved in patients' pathology could assist in explaining surgical approaches or techniques, while detailed AI‐generated graphics and videos could translate clinical documentation into easily understandable educational materials. Additionally, the concept of a “digital twin”—a digital replica of a patient created by AI through the collective inputs of clinical history, imaging studies, and wearable sensors—has enabled clinicians to tailor interventions with greater precision and assist patients in visualising their anticipated care trajectory [5, 25, 26, 34, 50, 51, 69, 77]. Through the manipulation of such models, physicians can educate patients on the pathophysiology of their condition and better illustrate the effect of proposed surgical treatment options. This was demonstrated in a recent randomised controlled trial where AI‐enabled decision aids led to improved decision quality and patient reported outcomes in patients with knee arthritis considering arthoplasty [39]. This level of education can improve clinical outcomes by fostering deeper patient engagement, aligning expectations, and encouraging treatment adherence, ultimately empowering patients to make informed decisions about their care.

### Generative medical event models

**Figure 3:**
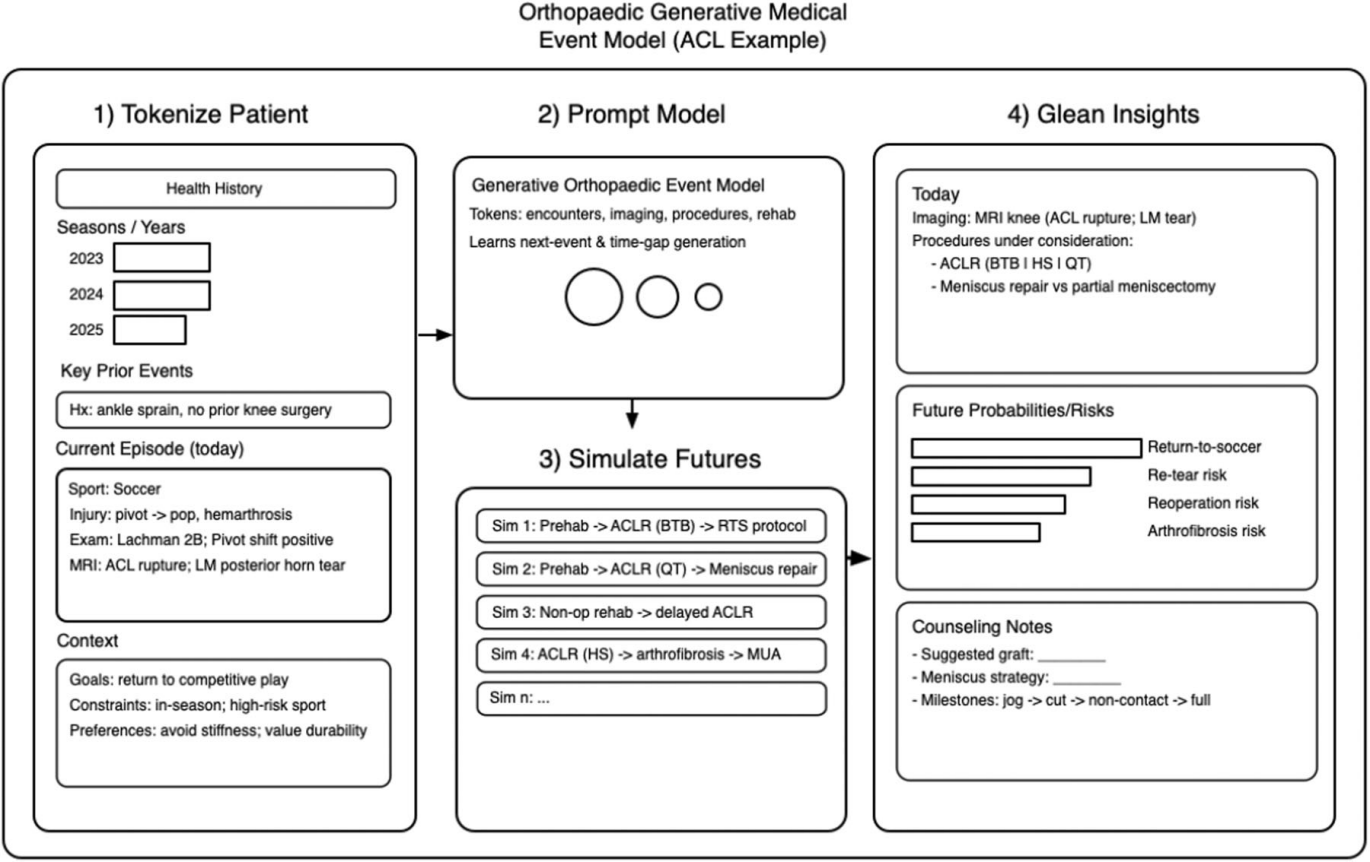
Example of an orthopaedic generative medical event model portraying a patient with a first time anterior cruciate ligament (ACL) rupture and lateral meniscus tear. Generative medical event models characterise a patient and simulate future outcomes based on large amounts of training data.

Rapid advancements in AI modelling may soon actualise the long‐standing goal of precise and personalised healthcare. Generative medical event models are an emerging form of individualised prognosis that incorporate spatiotemporal analysis of clinical datapoints in order to provide a framework of a patient's risk of select medical occurrences [72, 76]. Specifically, the creation of these models entails scaled pretraining with large volumes of clinical data to generate simulated timelines of probable medical events, including emergency department visits, inpatient admissions, and procedural interventions (Figure 3) [76]. When trained with sufficiently large datasets, these models can be applied to produce continually evolving predictions of a patient's likely disease course based on their unique medical history and contributing biopsychosocial factors. Accordingly, generative medical event models carry the potential to produce highly personalised and evidence‐based decision‐making frameworks that empower physicians and patients to create informed treatment plans and improve patient outcomes.

Early implementation of medical event models has proved promising, with training on expansive medical databases, such as EPIC's Cosmos dataset (EPIC Systems, Verona, WI), enabling exceptional predictive efficacy when applied to complex clinical data [72]. Namely, Cosmos Medical Event Transformer (CoMET) and Cosmos AI—a family of medical event models within EPIC's EHR platforms—have recently demonstrated their ability to produce accurate and efficient predictions of clinical events when trained on the more than 115 billion medical events contained within the Cosmos dataset. Specifically, utilisation of CoMET to predict anticipated medical encounters, disease‐specific outcomes, and plausible clinical diagnoses markedly outperformed similarly‐tasked models across an array of distinct pathologies [72]. While the full potential of these models remains to be seen, these early findings suggest that similar models may soon revolutionise a physician's decision‐making framework, and change how patients are counselled and monitored, thereby improving appropriate selection of surgical candidates and enabling preemptive interventions.

### Challenges and limitations

Despite the potential benefits of AI to clinical practice, there are a number of limitations that currently restrict its large‐scale implementation. Primary among these are concerns about patient safety and privacy, particularly as large datasets used to train AI models remain vulnerable to re‐identification of anonymized patient data when cross‐referenced with external sources [33, 44, 54]. Additionally, the integrity of AI models is dependent on the data they are trained on, allowing imbalanced or incomplete datasets to introduce algorithmic bias into decision‐making models that influence aspects of clinical care [15, 17, 36, 48, 53, 73]. These issues are compounded by questions surrounding accountability when adverse events occur, with uncertainty toward whether responsibility for AI‐guided decisions lies with developers, deploying institutions, or clinicians [10, 16, 66]. Furthermore, the absence of clear regulatory frameworks and reimbursement models for AI‐enabled tools limits enthusiasm for widespread integration [1, 56, 57]. Collectively, these challenges underscore the need for a transparent and responsible framework for AI advancement, one where physicians maintain clinical autonomy by using AI as a supportive, rather than a directive, tool (Table 1).

**Table 1:** Fact box.

Integration of artificial intelligence (A)I‐augmented vision systems may increase the breadth of information readily available to clinicians, allowing for objective assessments of gait patterns, deviations in limb alignment, and limitations to range of motion.
Smart examination rooms equipped with pressure‐ and motion‐sensitive technologies could enable physicians to leverage objective measurements to diagnose orthopaedic ailments and may improve the accuracy and utility of virtual encounters.
Clinical summaries and patient messaging generated by AI platforms may soon streamline clinical workflows to decrease administrative burden and allow more time for direct patient care.
Personalised education materials and visual aids, such as digital twin models, may improve patients' understanding of their unique pathology and enhance compliance with proposed treatment pathways.
Generative event models may soon alter decision‐making heuristics, provide anticipated medical trajectories, and improve individualised counselling in orthopaedic practices.
Physicians will likely come to share an increasingly symbiotic relationship with AI platforms, making it imperative that current and future orthopaedic practitioners become well‐versed in harnessing the capabilities of AI.

Specific to the applications explored above, technical, and financial barriers currently limit meaningful incorporation into clinical practice. For example, adoption of comprehensive, AI‐supported examinations rooms would require institutions to set aside or remodel existing spaces to allow for installation of an array of advanced diagnostic devices—each of which would incur considerable financial investments and require specialised personnel and data storage facilities for ongoing maintenance and software optimisation. Similarly, many of the platforms examined in this text would require ongoing technical advancement and access to robust training data sets to be able to effectively accomplish accurate and generalisable clinical analysis across diverse patient populations. Additionally, as many of these proposed applications are in the early stages of their development, an extensive process for achieving clinical approval and establishing appropriate regulations would likely be required before they become readily available for use. Consequently, without substantial institutional investment and coordinated oversight, the widespread integration of these technologies into orthopaedic practices remains a distant goal.

## CONCLUSIONS

Collectively, AI‐enabled technologies present enormous potential for redefining the delivery of orthopaedic care. AI‐augmented vision systems will increase the breadth of information readily available to clinicians, whereas smart exam rooms and automated clinical summaries may soon streamline clinical workflows to decrease administrative burden and allow more time for direct patient care. Personalised education materials and visual aids may improve patient understanding and compliance, with the aim of optimising patient outcomes. Generative medical/orthopaedic event models may soon alter decision‐making heuristics and improve patient counselling. While the widespread adaptation of AI into clinical practices is not without limitations, physicians will likely come to share an increasingly symbiotic relationship with these platforms throughout their continued evolution. As such, it is imperative that current and future orthopaedic practitioners become well‐versed in harnessing the capabilities of AI and continue to identify new avenues for such technologies to benefit clinicians and patients alike.

## AUTHOR CONTRIBUTIONS

All listed authors have contributed substantially to this work: Alexander Bouterse, James A. Pruneski, Felix C. Oettl, and Balint Zsidai performed literature review, Alexander Bouterse performed primary manuscript preparation. Editing and final manuscript preparation was performed by James A. Pruneski, Felix C. Oettl, Balint Zsidai, Michael T. Hirschmann and Kristian Samuelsson. All authors read and approved the final manuscript.

## ETHICS STATEMENT

None declared.

## CONFLICT OF INTEREST STATEMENT

KS is member of the Board of Directors of Getinge AB (publ) and medtech advisor to Carl Bennet AB. MTH is the Editor‐in‐Chief of KSSTA, and a consultant for Medacta, Symbios and Depuy Synthes.

## Data Availability

Data sharing not applicable to this article as no datasets were generated or analysed.
